# Infants’ sensitivity to emotion in music and emotion-action understanding

**DOI:** 10.1371/journal.pone.0171023

**Published:** 2017-02-02

**Authors:** Tik-Sze Carrey Siu, Him Cheung

**Affiliations:** 1 Department of Early Childhood Education, The Education University of Hong Kong, Tai Po, Hong Kong; 2 Department of Psychology, The Chinese University of Hong Kong, Shatin, Hong Kong; University of California, San Francisco, UNITED STATES

## Abstract

Emerging evidence has indicated infants’ early sensitivity to acoustic cues in music. Do they interpret these cues in emotional terms to represent others’ affective states? The present study examined infants’ development of emotional understanding of music with a violation-of-expectation paradigm. Twelve- and 20-month-olds were presented with emotionally concordant and discordant music-face displays on alternate trials. The 20-month-olds, but not the 12-month-olds, were surprised by emotional incongruence between musical and facial expressions, suggesting their sensitivity to musical emotion. In a separate non-music task, only the 20-month-olds were able to use an actress’s affective facial displays to predict her subsequent action. Interestingly, for the 20-month-olds, such emotion-action understanding correlated with sensitivity to musical expressions measured in the first task. These two abilities however did not correlate with family income, parental estimation of language and communicative skills, and quality of parent-child interaction. The findings suggest that sensitivity to musical emotion and emotion-action understanding may be supported by a generalised common capacity to represent emotion from social cues, which lays a foundation for later social-communicative development.

## Introduction

As adults we see one another as mentalistic agents whose behaviour is driven by intention, emotion, and belief. We thus routinely represent others’ inner thoughts and feelings so as to interpret and predict their action. Infant research using spontaneous-response tasks has established that such a mentalising ability emerges much earlier than we have thought (see [1] for a recent comprehensive review). The early development of mental state attribution suggests its fundamental role in social interaction. We can easily imagine how futile, or even embarrassing, an interaction could go if we fail to keep track of others’ mental states. Hence we constantly update others’ emotions and beliefs so that we can respond tactfully for maintaining positivity within groups [2].

Given that mindreading is essential to navigating the social world, one intriguing issue is how we may get access to others’ mind. Mental states are internal, private, and unobservable; we can but only infer them from overt behaviour. Prior work has revealed several types of information that infants use to make such inferences, such as an agent’s prior choice [3–6], eye gaze [7–8], emotional displays [9–10], and utterances [11–13]. Yet another cue to mind states possibly used by infants is music, which is conventionally regarded as a language of emotion conveying affective information in a unique way [14–15]. Basic emotions are assumed to be readily represented in music [16], which is made possible by manipulating basic acoustic (e.g., pitch, tempo, rhythm, loudness, timbre) and musical-system-specific cues (e.g., mode) [14,17–18]. This explains the ubiquity of infant-directed singing and speech in parent-infant bonding because the melodies effectively engage preverbal infants in emotion and intention sharing [19–22]. For instance, Rock, Trainor, and Addison [23] showed that mothers considered the goal of communication (to arouse or soothe the infants) and rendered the same song in different performance styles accordingly. The intended messages were well received by the 6-month-olds, who directed their attention inward when the song was sung as a lullaby but outward to the external environment when it was sung in a more playful style [23]. Four-month-olds who listened to consonant music also vocalised more and fretted less than those listening to dissonant music [24]. Therefore, caregivers typically capitalise on musical exchanges to communicate inner thoughts and feelings to their preverbal infants [25].

Because music is integral to parent-infant interaction, we argue that infants can draw on the affective information coded in music to represent others’ intention. Siu and Cheung [26] trained 15-month-olds on the association between various emotions and their accompanying facial expressions and body movements over three months, either with or without engaging the infants with music during training. Their findings showed that musical engagement not only promoted the infants’ sensitivity to musical expressions but also facilitated their understanding of the link between emotion and behaviour in a non-musical context. The authors argue that a sensitivity to the expressive power of music has a positive effect on the affective interpretation of everyday behaviour, and that this is trainable at 15 months.

In the present study we further examine if the link between sensitivity to music and affective understanding of behaviour develops spontaneously without explicit instruction and the timeline of such development. Specifically we ask three questions. First, we examine infants’ spontaneous sensitivity to emotion in music. While infants are sensitive to various musical dimensions such as pitch, consonance, rhythm, and metrical structures [18,21,27–31], there is a lack of robust evidence indicating their extraction of affective meaning from music [18,32]. In the present study, we tested 12- and 20-month-olds on their sensitivity to emotion behind music using a violation-of-expectation paradigm, in which the infants were presented with emotionally concordant (happy and sad music with happy and sad faces respectively) and discordant displays (happy and sad music with sad and happy faces respectively) on alternate trials. If the infants could identify the emotions behind the music, they would look longer at the discordant than concordant displays because the discordant displays violated their expectation of music-face consistency in emotional terms. Affective understanding of music was thus indicated by looking time differences between the concordant and discordant displays.

The second question we ask is whether infants at this age range are able to represent others’ emotion from facial-vocal expressions to predict their action. To test such emotion-action understanding, we adapted Phillips et al.’s [10] violation-of-expectation paradigm in which an agent posed either a happy or disgusted expression (counterbalanced across infants) towards one of two toys, followed by her subsequent grasping of them. Emotion-action understanding was operationalised as the longer looking times when the agent grasped the toy that had not received a happy expression or had received a disgusted expression, compared to the other toy.

The third question is whether emotional representation in music and non-musical emotion-action understanding are naturally correlated, and if they are, at what age the relationship would become observable. We hypothesise the relationship because both abilities may be supported by the same underlying, more generalised capacity to represent others’ emotional states, which is fundamental to the infant’s early social development [26]. This generalised capacity is assumed to underpin non-musical facial-vocal expression, goal-directed action, as well as musical expression.

## Method

### Participants

A total of 57 typically developing 12- and 20-month-old infants (27 boys) were tested. Parents responded to advertisements posted on local online parent-child forums and brought their infants to the laboratory to participate. The data from 7 infants were dropped from analysis because of their extreme fussiness and crying during test (4), or inter-observer reliabilities in looking time coding lower than 0.8 (3). The final sample thus included 24 12-month-olds (11 boys, mean age = 12.10 months; *SD* = .18 month) and 26 20-month-olds (14 boys, mean age = 20.39 months; *SD* = .14 month). All the infants were full-term and had no known perceptual, psychological or linguistic abnormalities. They were all ethnically Chinese and were born and brought up in Cantonese-speaking families in the city of Hong Kong. Parents’ written informed consent on infants’ participation was sought prior to testing. The infants and their parents received a certificate for their participation. This research and the written consent form were approved by the Ethics Committee, the Social Science Panel, the Chinese University of Hong Kong.

### Materials and procedure

We first asked the parents to fill in a demographic questionnaire before proceeding to the testing session. Information such as family income and daily parent-child interaction quality (e.g., language for everyday interaction) was obtained. Testing consisted of two tasks, namely, sensitivity to emotion in music and emotion-action understanding, which were separated by a period of free play. The infants were randomly assigned to one of the two testing orders.

#### Sensitivity to emotion in music

The sensitivity to emotion in music test used a 2 x 2 mixed design with age as the between-subject factor and music-face emotion consistency in the test trials as the within-subject factor. Each infant was exposed to both the happy and sad emotion in the familiarisation trials prior to test. The dependent measure was infant looking time.

We followed prior studies and chose a selection of instrumental music excerpts from the classical repertoire which had been reliably characterised as happy or sad music [32–35]. Instrumental instead of vocal music was used because of two reasons. First, we are interested in the derivation of emotion from music as an abstract form which is void of any non-musical carriers of emotion, such as the human voice. Second, because we seek to correlate infants’ sensitivity to musical emotion (sensitivity to emotion in music) with their interpretation of non-musical emotion expressed via facial-vocal means (emotion-action understanding), it is important not to involve the human voice in the music test so as to avoid bringing in a common vocal element that would make any correlation between the two tests trivial. We asked a group of undergraduates who had no formal music training to rate on the extent to which each of these 1-minute excerpts communicated the emotions of happiness and sadness, using a 9-point likert scale. Finally, Saint-Saëns’ *Le Carnaval des Animeaux* and Ravel’s *Le Tombeau de Couperin* were selected as happy music whereas Chopin’s *Nocturne Op*. *9 No*. *1* and Bruch’s *Kol Nidrei* were selected as sad music for subsequent use because they received the most extreme happiness and sadness ratings, respectively. The mean tempos of the happy and sad music were 170 and 60 beats per minute, respectively. This is consistent with previous analyses showing that tempo is an important cue contributing to our experience of musical emotion [17]. Hence, in the present study, tempo could also be used by the infants as a prominent cue to musical emotion.

Twelve Cantonese-Chinese adult females were recruited as actresses to pose dynamic facial expressions of happy and sad feelings. They were first shown universal prototypes of happy and sad faces from the Facial Action Coding System (FACS) [36]. The distinctive facial features for the respective emotions were highlighted and explained to them. They were then asked to simulate a happy and a sad version of the following episode in Cantonese: “Here’s your milk. Drink it all up. Don’t spill any. Oh look, you got some on the tray. Don’t play in it. I’ll get a sponge and clean it up.” Previous research showed that preschoolers and adults were able to discriminate the emotions behind the happy and sad renditions of this script, even with a muted version [37]. We used dynamic affective displays because young infants were shown to be insensitive to emotional information depicted in still faces [38]. We videotaped the actresses from their shoulders up against a white wall under normal lighting while they were rendering the script in a happy or sad manner. These simulated episodes were presented to a group of undergraduates who rated them on the degree of happiness and sadness. The two happy and two sad videos receiving the most extreme ratings were selected for subsequent use. The mean articulation rates of happy and sad videos were 2.45 and 2.07 syllables per second, respectively.

The experimental setup and procedure were previously described in detail in Siu and Cheung [26]. The infants sat on their parents’ lap during the testing session. They faced a curtained stage (40 cm high x 108 cm wide) enclosed in a display booth (180 cm high x 116 cm wide x 78 cm deep) at a distance of 60 cm. Parents were instructed to listen to masking music via headphones and limit interaction with their infants during the procedure. On the stage stood a 24-inch colour monitor which presented the actress’s dynamic facial expressions during the test. The affective displays were concealed by a curtain before the onset of test and between trials. Musical excerpts were played by a computer connected to loudspeakers on both sides of the display booth. A digital camcorder was mounted below the stage from an angle optimal for coding to videotape infant behaviour throughout the testing session. The camcorder was hidden by a curtain with only its lens protruding. Two independent observers, who were naïve to the type of test events to which the infants was exposed and could not see the experimental display, watched this visual output on a monitor in an adjacent room and coded the infants’ attention to the display on the spot. They were trained to keep depressing a button when the infant was fixating on the display and release it when the infant looked away. A computer programme captured the time lengths of these button presses to work out the infants’ looking times online. Trial presentation was controlled by the observation of the primary observer, whereas the judgement of the secondary observer was used to calculate an inter-observer reliability.

Testing was modelled after the violation-of-expectation procedure used by Luo and colleagues (e.g., [39–40]), comprising a familiarisation and a test phase. The three familiarisation trials followed either a happy-sad-happy or sad-happy-sad sequence, counterbalanced across infants, so that each infant was exposed to both emotions. In a happy trial, happy music was played to the infant via loudspeakers and after 10 seconds of listening the closed curtain was drawn so that the monitor was visible to the infant. The monitor presented a silent video clip which showed an actress posing a happy facial expression while the music kept playing. A sad trial followed the same procedure except that sad music and a sad facial display were used. For any familiarisation trial the emotion behind the music was always congruent with the emotion expressed by the actress in the video clip. The same happy and sad music were used and the same actress was featured throughout familiarisation. A familiarisation trial ended when the infant looked away for 2 consecutive seconds after having looked for at least 5 cumulative seconds, or when 30 cumulative seconds had elapsed.

Two consistent events alternated with two inconsistent events in the test phase. In both types of test events, the infant first listened to happy or sad music (counterbalanced across infants) for 10 seconds. After that, while the music kept playing, the infant was shown an actress whose affective expression either matched (in a consistent event) or mismatched the emotion behind the music (in an inconsistent event). The musical excerpts and the actress in the test trials were different from those in familiarisation. A test trial ended when the infant looked away for 2 consecutive seconds or had looked for 60 cumulative seconds without looking away. Presentation order of the consistent and inconsistent test events (consistent-first or inconsistent-first) was counterbalanced across infants. The whole sensitivity to emotion in music procedure took about 10 minutes.

The present violation-of-expectation paradigm differs from the habituation procedure used by Flom and colleagues [33,41] in one critical aspect, which is the relationship between the familiarisation (or habituation) and the test trials. In Flom et al.’s [33,41] procedure, discrimination was indicated by release from habituation (response recovery) due to the presentation of new stimulus items at the test stage. Such release was possible only if the habituation trials prior to test had significantly decreased the infants’ responding to the old stimulus item. In contrast, the present violation-of-expectation test was based on surprise, not release from habituation [39–40]. In the test trials, music-face incongruence was assumed to be inherently more surprising than music-face congruence, and thus incongruence would always attract more attention from the infant, independent of familiarisation. Note that the music and actress in familiarisation were different from those in the test trials. The three familiarisation trials were not to induce habituation but were only to familiarise the infants with the test procedure, so that they were better prepared for the test trials. The non-surprisingness of music-face congruence at test was not assumed to be a result of familiarisation. Hence, while the habituation trials were integral to Flom et al.’s [33,41] procedure, our familiarisation trials did not directly contribute to the expected effect in the test trials. For this reason, we did not apply any criteria for habituation to the present procedure.

#### Emotion-action understanding

The emotion-action understanding test used a 2 x 2 mixed design with age as the between-subject factor and emotion-action consistency in the test trials as the within-subject factor. The random grouping of the infants into a “happy” and “disgust” group was not an independent factor; “happy” and “disgust” were only two situations in which emotion-action consistency and inconsistency could occur (see [10,42] for an argument for this design). Hence, in analysis the data from the two groups were pooled together with regard to emotion-action consistency. The dependent measure was infant looking time.

The infants were seated on their parents’ lap and saw events which took place on the same curtained stage. In this task, the stage held two identical toy bears in different colours side by side 48 cm apart. An actress sat in a chair behind the stage so that her face and upper body were visible to the infants when the curtain opened. A camcorder was positioned below the stage for the trained observers to code infants’ visual fixations at the display during test. To minimise parental interference, parents were asked to remain neutral and not to interact with their infants throughout the procedure.

The emotion-action understanding test was modelled on the paradigms used by Phillips et al. [10] and Vaish and Woodward [42]. The procedure was reported in detail in Siu and Cheung [26]. We randomly assigned the infants to a happy or disgust condition, each consisting of three familiarisation and four test trials. Familiarisation started when the curtain opened, revealing an actress with a neutral expression behind a stage on which two toy bears in different colours rested side by side. After the infant had been attending to the display for 2 seconds, the actress started to show different emotions depending on the condition. In the happy condition, the actress smiled, looked at one of the two bears, and said excitedly “Oh! Look at that teddy bear!” The actress held this facial expression and visual regard for 5 seconds before the curtain was closed. When the curtain reopened, the infant saw the actress looking at and grasping the same bear which she had previously regarded with excitement and interest. In familiarisation, the emotion expressed by the actress was always consistent with her grasping action. A familiarisation trial ended when the infant looked away from the display for 2 consecutive seconds, or when 30 cumulative seconds of looking had elapsed.

During the test phase, the infants were shown two consistent and two inconsistent test events in alternation. In a consistent event, after the infant had looked at the display for 2 seconds, the actress emoted positively towards the other bear, the one not regarded and held in familiarisation, for 5 seconds before the curtain was closed. When the curtain reopened, the actress reached for this same bear. Similar to familiarisation, the actress’s reaching action corresponded with her prior expressed emotion towards the bears. In an inconsistent event, the actress looked and smiled at the bear she had regarded and held during familiarisation; however, she then grasped the other bear. Because the actress did not approach the bear which she previously had felt excited about, this event sequence was considered as inconsistent. A test trial ended when the infant looked away for 2 consecutive seconds, or had looked for 60 cumulative seconds.

Infants in the disgust condition first saw an actress lowered her brows, retracted her neck and said “Yuck! Look at that teddy bear!” towards one of the two bears (Bear A) with distaste during familiarisation. When the curtain reopened, the actress held the other bear which had not been negatively emoted about before (Bear B). After three familiarisation trials, two consistent and two inconsistent test events alternated. In a consistent event, the actress emoted negatively towards Bear B and subsequently grasped Bear A; in an inconsistent event, she emoted negatively towards but also grasped Bear A. The test trials ended when the infant looked away from the display for 2 consecutive seconds, or had looked at the display for 60 cumulative seconds. Locations of the toy bears, the bear grasped in familiarisation, and presentation order of the consistent and inconsistent test events were counterbalanced across infants. The emotion-action understanding test took about 10 minutes.

#### Reliability coding

We used a reliability coding scheme that was described in detail in Siu and Cheung [26]. Both the primary and secondary observers recorded online the infants’ attention to the display during the familiarisation and test trials. To assess their agreement in coding, a computer programme which tracked infant looking times divided each second into 10 equal intervals. Within each 100-ms interval, the programme compared the two observers’ binary judgements on whether the infant was attending to the display. Inter-observer reliability was calculated by dividing the number of intervals in which the two observers agreed by the total number of intervals for each infant. We discarded recordings with inter-observer reliabilities lower than .80 because of judgement inconsistency between the two observers. Overall reliability for the 12- and 20-month-olds were .90 and .93, respectively.

To ascertain that the actress’s live acting in the emotion-action understanding test did not vary systematically across the experimental conditions, we asked two naïve raters to watch videos showing the actress’s facial-vocal expressions and to judge whether the acting was from the consistent or inconsistent test events. For the 12-month-olds, the overall accuracies were 48% and 54% for the consistent and inconsistent events, respectively; for the 20-month-olds, the corresponding accuracies were 57% and 51%. Such near-chance discrimination did not suggest an actress bias in enacting the test events.

#### Language and communicative skills development

We also ask the parents to complete the Cantonese version of the MacArthur-Bates Communicative Development Inventory (CCDI) [43–44]. As reported previously in Siu and Cheung [26], CCDI is a standardised parental-report instrument which tracks infants’ and toddlers’ developing language and communicative abilities with local norms [43–44] and excellent internal reliabilities (Cronbach’s alpha, Infant form: .97; Toddler form: .99). The Words and Gestures Forms (Infant form) were used with the 12-month-olds to record the words they understood and produced, and also the communicative and symbolic gestures they attempted or completed. We administered the Words and Sentences Forms (Toddler form) to the 20-month-olds where the parents were asked to document the words or multi-word utterances their children understood and used, and to provide three examples of the longest sentences their children produced. We used the short form with both groups because it was more time-efficient and was adequately correlated with the full form (*r* = .98 for vocabulary comprehension, and *r* = .97 for vocabulary production; [45]). The maximum scores of the Infant and Toddler forms were 205 and 138, respectively.

## Results

### Sensitivity to emotion in music

Preliminary examination revealed no significant effects of gender, socio-economic background, the emotion first presented in familiarisation, and the presentation order of test events on the infants’ looking times. These variables were thus collapsed in all subsequent analyses.

#### Familiarisation

The 12-month-old infants looked for an average of 22.82s (*SD* = 7.71s), 15.59s (*SD* = 9.86s), and 13.59s (*SD* = 8.82s) on the first, second, and third familiarisation trial, respectively. We conducted a repeated-measures Analysis of Variance (ANOVA) and found a significant decline in attention across the familiarisation trials, *F*(2, 21) = 8.48, *p* = .002. The mean looking times for the 20-month-olds on the first, second, and third familiarisation trial were 27.12s (*SD* = 6.33s), 22.92s (*SD* = 9.03s), and 18.72s (*SD* = 10.74s), respectively. A repeated-measures ANOVA showed that the overall looking time decrement was significant, *F*(2, 24) = 8.43, *p* = .002. These results suggested that both groups of infants adequately received the information presented in familiarisation.

#### Test events

**Fig 1:**
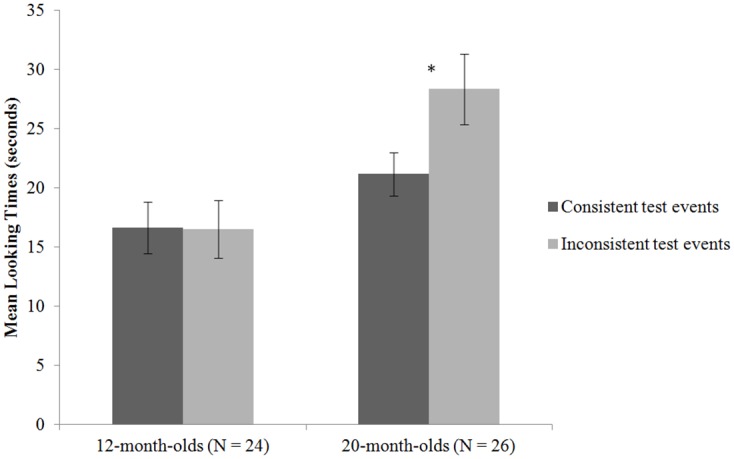
Infants’ mean looking times in the sensitivity to emotion in music test.

Fig 1 summarises the 12- and 20-month-olds’ looking times in the test events. We averaged the looking times from the two trials in each condition and compared the conditions. Such within-condition averaging across trials provided a more reliable estimate of the infants’ attention than relying on a single trial [10,42]. The average looking times were submitted to a 2 x 2 mixed-design ANOVA with test event (consistent vs. inconsistent) as the within-subject factor and age (12 vs. 20 months) as the between-subject factor. Because there were 26 20-month-olds but only 24 12-month-olds, we dropped two infants with the lowest inter-observer reliabilities from the older group so as to achieve equal sample sizes for the two age groups, which was important for ANOVA. The interaction effect was significant, *F*(1, 46) = 4.47, *p* = .04. While the 12-month-olds showed similar looking times in the consistent (*M* = 16.62s, *SD* = 10.79s) and inconsistent test events (*M* = 16.48s, *SD* = 11.98s; *t*(23) = .04, *p* = .97.), the 20-month-olds looked significantly longer in the inconsistent (*M* = 29.02s, *SD* = 15.16s) than consistent events (*M* = 20.27s, *SD* = 9.14s; *t*(23) = -3.10, *p* = .005; Fig 1). We also performed a chi-square test and found that the proportion of infants who looked longer at the inconsistent test events differed significantly between the two age groups, *χ*^2^ (1, 48) = 8.39, *p* = .004. Only 8 out of the 24 12-month-olds looked longer at the inconsistent than consistent test events, while 18 out of the 24 20-month-olds did so.

### Emotion-action understanding

No data were discarded because of inappropriate acting. Preliminary analyses indicated no significant correlations between the infants’ looking times and their gender, socio-economic status, locations of the toy bears, the target toy that was reached for in familiarisation, and the type of the test event presented first. Looking times did not differ between the emotion conditions (happy and disgust). Hence, we collapsed all these factors in the subsequent analyses.

#### Familiarisation

The average looking times of the 12-month-olds in the first, second, and third familiarisation trial were 24.17s (*SD* = 7.50s), 19.96s (*SD* = 10.25s), and 17.13s (*SD* = 9.82s), respectively. These looking times were submitted to a repeated-measures ANOVA and the decrement across the familiarisation trials was significant, *F*(2, 22) = 4.17, *p* = .029. The 20-month-olds’ attention to the familiarisation events also declined significantly from 26.00s (*SD* = 7.34s) in the first trial to 18.63s (*SD* = 10.57s) in the last trial, *F*(2, 24) = 5.42, *p* = .011. The overall drop in the infants’ attention suggested that they were actively encoding the event sequences.

#### Test events

**Fig 2:**
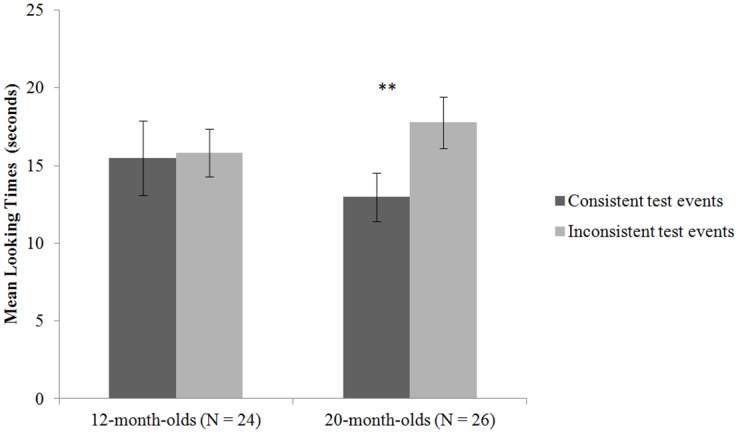
Infants’ mean looking times in the emotion-action understanding test.

Looking times from the two test trials in each condition were averaged and the conditions were compared (Fig 2). The average looking times were analysed by a 2 (test event: consistent vs. inconsistent) x 2 (age: 12 vs. 20 months) mixed-design ANOVA. Because there were 26 20-month-olds but only 24 12-month-olds, we dropped two infants with the lowest inter-observer reliabilities from the older group so as to achieve equal sample sizes for the two age groups, which was important for ANOVA. The two-way interaction effect was significant, *F*(1, 46) = 4.77, *p* = .034. The 12-month-olds showed no significant looking preference for either the consistent (*M* = 15.48s, *SD* = 11.76s) or inconsistent test events (*M* = 15.83s, *SD* = 7.48s; *t*(23) = -.18, *p* = .86). In contrast, the 20-month-olds looked significantly longer at the inconsistent (*M* = 18.07s, *SD* = 8.11s) than the consistent test events (*M* = 12.45s, *SD* = 7.60s; *t*(23) = -3.92, *p* = 001). In addition, the proportion of infants who looked longer at the inconsistent displays varied across the age groups, *χ*^2^ (1, 48) = 4.46, *p* = .035. Only 12 12-month-olds looked longer at the inconsistent than consistent events, compared to 19 20-month-olds who showed such a looking preference.

### Relation between sensitivity to emotion in music and emotion-action understanding

We further investigated the extent to which performance on the sensitivity to emotion in music test was associated with that on the emotion-action understanding test. For the emotion-action understanding test, we followed Wellman, Phillips, Dunphy-Lelii, & LaLonde’s [46] measure and computed a difference score for each infant by taking the difference in looking time between the consistent and inconsistent test events. We assumed that this score reflected their awareness of inconsistency between the actress’s expressed emotion and her subsequent action. For the music test, we derived a similar difference score indicating the infants’ sensitivity to how much the actress’s facial expression matched the music in emotion terms. One basic assumption behind the difference score was that looking times from the consistent trials provided a baseline measure of the infant’s spontaneous attention to “normal” emotional events, when what was read from the face was consistent with the music that was heard (music test) and the action that was seen (emotion-action test) in emotional terms. The next assumption was that any looking time deviation from this baseline induced by the inconsistent trials provided a quantitative measure of how much the infant was aware of the “abnormality”, when what was read from the face contradicted the music that was heard (music test) and the action that was seen (emotion-action test) in emotional terms. With these assumptions, the difference score method has been used in previous studies on infants’ understanding of intentional action [47–48]. In the present study, a simple correlation analysis revealed a significant association between the difference scores from the music test and the emotion-action test for the 20-month-olds, *r*(26) = .52, *p* = .006, but not the 12-month-olds. For the 20-month-olds, the association remained significant even after the effects of language and communicative skills, family income, and quality of daily parent-child interaction were partialled out, *r*(23) = .56, *p* = .02. These variables themselves did not correlate with the sensitivity difference scores from the music test nor the emotion-action understanding test (all *p*s > .10).

## Discussion

We obtained three findings in the present study. First, 20-month-olds, but not 12-month-olds, were sensitive to emotional incongruence between musical and facial expressions, suggesting an awareness of emotion behind music. Second, these 20-month-olds were also able to use an actress’s emotional facial-vocal display to predict her subsequent grasping action. The 12-month-olds failed to do this. Finally, sensitivity to emotion in music was associated with emotion-action understanding for the 20- but not 12-month-olds. This association in the older infants has a certain degree of specificity because neither the music test nor the emotion-action test sensitivity score correlated with any of the more general variables of family income, parental estimation of language and communicative skills, and quality of parent-child interaction.

The present results extend prior findings that children start to interpret the emotions expressed in music at 4 to 5 years [32,49–53]. To test this understanding, past research has used an elicited-response task in which young children explicitly point to a schematic face that matches the emotion behind the music heard. In contrast, the present study adopts a more implicit paradigm that capitalises on infants’ spontaneous increased attention to expectation-violating events as indicated by increased looking. We think that this paradigm provides a sensitive measure of the infant’s awareness of emotion in music because it relies on spontaneous looking and does not require the participant to make explicit decisions, which may be affected by multiple extraneous factors such as understanding of task demand. The paradigm particularly suits infants and younger children who have limited cognitive control to solve focused tasks calling for explicit decisions. With this more sensitive paradigm, therefore, we argue that the first evidence for an affective interpretation of music is obtainable during the second year of life.

Using a habituation looking procedure, Flom and colleagues [33,41] demonstrated that infants as young as 5 months could distinguish happy from sad musical excerpts. While such discrimination indicates the infants’ sensitivity to the acoustic contrasts between happy and sad music (e.g., tempo, mean pitch and range), there was no evidence that they were able to read the emotional content of the music and associate it with other non-musical expressions of emotion. In our paradigm we presented the infants with happy and sad music along with emotionally congruent or incongruent facial expressions. The infants had to interpret the music in emotional terms in addition to merely picking up the acoustic cues before the music-face discordant displays could surprise them. In other words, discrimination of acoustic cues is necessary for but does not guarantee the kind of emotional interpretation that is required by the present music test. Hence we consider the present cross-modal paradigm a more vigorous test of infants’ affective interpretation of music, which is richer and more sophisticated than the mere discrimination of acoustic cues [33,41]. Nawrot [32] similarly presented 5- to 9-month-olds with cross-modal, music-face emotional displays but failed to find evidence for a general understanding of musical emotion, which concurs with our lack of results with the 12-month-olds. Taken together, our findings confirm that infants before their first birthday do not yet fully appreciate the emotional implications of music; we are able to see indications of this capacity only at 20 months.

Repacholi and Gopnik [54] found that 18-month-olds, but not 14-month-olds, were able to read the experimenter’s emotional expression and offer her the food she desired. Similarly, our results from the emotion-action understanding test showed that the 20-month-olds could use the actress’s emotional displays to infer her subsequent action. This understanding, however, was not evident in the 12-month-olds. The finding supports Vaish and Woodward’s [42] conclusion about the emergence of emotion-action understanding in two ways. First, we replicated their result that infants younger than 14 months failed to use emotive cues, including happy and disgusted expressions, to anticipate others’ behaviour. Second, we found that such emotion-action understanding was in place at 20 months, which corroborates their speculation that infants interpret behaviour in terms of emotional states only after 18 months [42]. We argue that compared to early social referencing, the relatively late development of emotion-action understanding may have to do with its more complicated motivation. In social referencing, the affective expressions of significant others serve to inform the infant about what emotion should be adopted and what to do next, particularly when he/she is confronted with ambiguous stimuli [55–56]. Such emotion adoption is straightforward, self-serving, and less interpretive. On the other hand, emotion-action understanding entails socially oriented inferencing about others’ inner motivational states for the prediction of their future action. Because this understanding involves sophisticated emotional interpretation and representation in reasoning about others’ behaviour, it is not surprising that infants start to grasp it only relatively late in the second year.

Further to Siu and Cheung’s [26] training results in the range from 15 to 18 months, the present study showed that 20-month-olds’ sensitivity to musical expressions was spontaneously related to their emotion-action understanding. Yet this relationship did not seem to have anything to do with more general language and communicative skills, family income, and quality of parent-child interaction. We argue that this specific association is supported by a common capacity to represent emotions from social cues underlying both abilities. In the present context, the cues in question are the music and facial-vocal expressions in the music and non-music task, respectively. In the music task the older infants were surprised by the mismatch between the music cues and the accompanying facial expressions which indicated emotion, whereas in the non-music task they were surprised by a lack of correspondence between the facial-vocal expression cues and the subsequent action. Emotions can be expressed in many ways. Prior work has predominantly studied infants’ attention to facial expressions in inferring others’ emotional states [9–10,42]. Yet preverbal infants and their caregivers also rely on musical exchange to communicate feelings and thoughts. Therefore, in addition to facial expressions, we think that infants also attend to music expressions as cues to others’ emotions. Our results with the 20-month-olds support this hypothesis. Furthermore, the infants who were able to read the emotion in music were also more likely to use the actress’s facial expression to predict her action. Apparently, these two abilities similarly call for an abstraction of affective meaning from social symbols (i.e., music and face). Perhaps once this broader affective awareness or skill starts to develop, the infant is encouraged to quickly identify a range of cues to further support an expanding sphere of communication in a flexible way. To fully examine this possibility, we need to systematically catalogue and compare infants’ understandings of different social-emotional cues at different developmental stages (e.g., eye gaze, body movement, facial, vocal, and musical expressions).

Emotion is an important driving force behind behaviour. In successful communication, we must constantly pick up emotive cues to infer and update ourselves on others’ emotional states. Such emotional representation helps us modify our behaviour tactfully, sparing us from unsatisfactory and even harmful interaction. The present study demonstrates that infants at 20 months are able to decode musical expressions and use facial expressions to reason about behaviour. These two abilities appear to be subserved by a facility for representing and interpreting social-emotional cues, which prepares the infant for complex social interaction.

## Supporting information

S1 Dataset Infants’ looking times in the current study. (SAV)
